# Effects of Plant Extracts on Growth Promotion, Antioxidant Enzymes, and Secondary Metabolites in Rice (*Oryza sativa*) Plants

**DOI:** 10.3390/plants13192727

**Published:** 2024-09-29

**Authors:** Ei Ei, Hyun Hwa Park, Yong In Kuk

**Affiliations:** Department of Oriental Medicine Resources, Sunchon National University, Suncheon 57922, Republic of Korea; eieitza@gmail.com (E.E.); camelia9720@nate.com (H.H.P.)

**Keywords:** antioxidative enzyme, growth promotion, plant extracts, rice, secondary metabolites

## Abstract

Plant extracts are widely used in sustainable agriculture practices to enhance crop production and reduce chemical usage in agriculture. This study employed several extraction solutions of various plant extracts to synthesize planting and spraying strategies, assess the persistence efficacy of rice, and investigate the influence of selected water extracts on secondary chemicals at different rice planting stages. Among 17 water extracts that were evaluated on rice seeds, 7 were enhanced to align with the lengths of rice roots 50–70% and shoots 40–50%. The analysis of extraction, spraying, and planting experiments revealed that water extracts, soil application, and transplanting were the most efficient methods for stimulating rice growth, especially 0.1 and 0.5% concentrations. The efficacy of the extracts remained intact also after 14 days of treatment. This study showed that photosynthesis and antioxidant activities may play crucial roles in plant growth. Rice growth stimulation has been linked to photosynthesis, flavonoid contents, and antioxidant enzymes, providing a balanced supply of nutrients for plant growth. Among all tested water extracts, *Psidium guajava*, *Aloe vera*, *Allium sativum*, and *Medicago sativa* extracts can be used to promote plant growth in organic farming.

## 1. Introduction

Rice is the staple food for approximately one-third of the world’s population. Rice is an economically important food crop with nutritional diversification and helps in poverty alleviation. About 90% of the world’s rice is grown and consumed in Asia [1]. In 2023, South Korea’s rice cultivation area was 0.71 million hectares, with a production rate of 3.6 million metric tons, as to World Agricultural Production [2]. Nevertheless, the world’s rice production still has space for improvement through increasing land productivity and raising its yield potential. Sustainable agriculture is a crucial global issue [3]. The efforts should be oriented towards minimizing the input costs, as well as the reliance on chemical fertilizers and pesticides, the misuse of which may pose many hazards to human life and the environment [4].

Alternative and sustainable techniques for overcoming these challenges are thus thoroughly studied [5,6]. Several strategies have been proposed, among which organic products called biostimulants are the most investigated and promising products to make agriculture more sustainable [5,7,8,9,10,11]. Biostimulants could amend a plant’s physiological processes to improve nutrient absorption and optimize its consumption [12]. Plant extracts have recently been extensively researched as a practical strategy for improving the sustainability of crop production, particularly for the generation of biostimulants [3].

Aloe leaf extract, especially at the highest concentration (40 mL/L), significantly increased the plant height, number of leaves, number of branches, yield, and essential oil percentage, as well as enhancement of the leaf anatomical structure [13]. A study on the cucumber plant noted [14] that the foliar application with garlic extract by a concentration of 2.5 mL L^−1^ led to a significant increase in the length of the plant, the number of leaves and foliar area, and the content of total chlorophyll in the leaves, the proportion of the nodes and total soluble solids. Guava leaves and neem seed waste compost had a better effect on the vegetative growth parameters of Temulawak, such as plant height, stem diameter, and leaf length and width [15]. The aqueous extracts of alfalfa have a significant effect on root length and yield in beetroot [16].

Korea has approved 1642 eco-friendly agricultural products for enhancing soil, growth stimulation, and controlling diseases and insects, including 45 plant extracts [17]. Korean agriculture books dating back to the 1920s describe farming systems that emphasize the need to use readily available natural resources to promote growth [18]. These traditional organic methods can contribute to the advancement of the current organic farming technologies. However, several agricultural organic materials utilized in traditional farming have not been adequately studied for their impact on growth promotion [19].

The number of studies attempting to determine the efficacy and potential of plant extracts has increased, but the current knowledge is still very limited to biological systems under learning, leaving aside the production systems and all potential applications, resulting in gaps that require further research. In addition to the application of plant extracts, it is necessary to understand how and where research in this area has been conducted and disseminated to provide useful information for planning, conducting, and publishing future research on the use of plant extracts in agriculture, with a focus on seedlings. The research hypothesis confirmed that less knowledge of plant extracts promotes growth physiology and chemical characteristics in rice plants.

The main objectives of the present study were to determine the effect of various water extracts on the growth of rice to investigate the effect of planting and spraying methods on the growth of rice treated with various plant extracts from water, ethanol, and boiling water extracts; to determine the effect of extract efficacy persistence, and to study the effect of selected water extracts on the growth of rice sprayed once on different sowing days, and to analyze the influence of selected water extracts on secondary metabolites and antioxidant enzymes at different rice growth stages.

## 2. Results and Discussion

### 2.1. Effect of Water Extracts Obtained from Different Agricultural Materials on the Growth of Rice

In this study, the growth promotion rates of rice were assessed using 17 water extracts from 15 different plant materials at concentrations of 0.05, 0.1, 0.5, and 1% in Petri dish bioassays (Table 1). All tested extracts boosted shoot length by 10–50%, root length by 10–70%, and seedling length (shoot + root) by 10–50%. Shoot length was found to be 40–50% in three (*P. guajava*, *A. vera*, *G. max* stem) of the 17 water extracts. Root length was significantly increased by 50–70% in two (*P. guajava* and *A. sativum*) of the 17 water extracts. Seedling length increased by 40–50% in four (*P. guajava*, *A. sativum*, *G. max* stems and leaves) of the 17 water extracts tested. The bioassay results indicated that *P. guajava*, *A. vera*, *A. sativum*, *M. sativa*, *A. tuberosum*, and *G. max* (leaves and stems) had the highest rate of rice growth. The compost from guava leaf extract gives positive benefits for vegetative growth [15]. In other studies [19], Chinese chive, soybean leaf, and soybean stem extracts induced a 31–45% increase in shoot fresh weight of lettuce compared with control plants. The treated eggplants demonstrated enhanced growth and physiological responses correlated with the frequency of aqueous garlic extract application and growth stage of the plants, respectively [20]. Foliar spraying with *A. vera* extract improved the growth and yield of sweet basil [21]. Furthermore, in vitro, bioassays showed that 0.05, 0.1, and 0.5% doses improved rice growth among concentrations. Regardless of the two metrics, the rice root length had a higher growth rate percentage than the rice shoot length in this study. Previous studies have shown that enhanced root activity can be influenced by root growth promotion in *Arabidopsis* [22]. Promoted root growth further facilitates the uptake of nutrients from the rhizosphere, thus enabling plants to accumulate incremented doses of water and soluble nutrients, subsequently improving plant growth [23].

### 2.2. Effect of Selected Extracts by Different Extraction, Spraying and Planting Methods on Rice Growth

In the Petri dish assay, the extracts (*P. guajava*, *A. vera*, *A. sativum*, *M. sativa*, *A. tuberosum*, *and G. max* (stems and leaves)) from the three extraction methods had a positive effect on rice growth at low concentrations compared to the control (Table 2). The reason for this was that the excluded extracts inhibited the growth of rice seedlings by 1% compared with the control. These results indicate the allelopathy of garlic, where low concentrations were growth-promoting, while higher concentrations inhibited the growth of the plants in question [24]. Aqueous garlic extract consists of organosulfur compounds, in particular, “allicin”, which is a strong antioxidant [25] and can inhibit the growth of recipient plants at higher concentrations [26]. Of all the treatments, the extracts of *P. guajava* and *M. sativa* had the greatest effect on rice growth for all extraction techniques and concentrations.

In the direct seeding and transplanting methods (Table 3), the results showed a significant difference in plant height and shoot fresh weight of rice seedlings when using the water extract method compared to the other methods used in this experiment. Results revealed in [14] that the water extract of the garlic plant contains 30% carbohydrates and is rich in the elements of potassium, iron, magnesium, phosphorus, thiamine, riboflavin, niacin, and ascorbic acid in addition to volatile oils. This study showed that soil application was more effective in promoting rice growth than foliar treatment for all extracts in the transplantation method. When rice seedlings were treated with the lowest concentrations (0.05, 0.1, and 0.5%) of these extracts, plant height and shoot fresh weight increased. However, there were no significant differences in the shoot fresh weight of rice seedlings between treatments at 7 and 14 DAT. In another study, the effect of biostimulants was found to vary depending on species and variety. Environmental factors, dosage amount, and timing of application were also found to influence the effectiveness of extract applications [27].

### 2.3. Effect of Persistence of Four Selected Extracts on Rice Growth

In this study, *P. guajava*, *A. vera*, *A. sativum*, and *M. sativa* were selected based on the results of a previous study (data not shown). For the water extracts, the results of shoot fresh weight stimulation showed that the Hopyeong variety had a significant difference in shoot fresh weight of direct seeding methods, ranging from 30 to 48%, whereas the Saenuri variety had a significant difference ranging from 35 to 48%. The fresh weight of the shoot was stimulated by the ethanol extracts by 28–38% for the Hopyeong variety and 30–35% for the Saenuri variety. In the transplanting trials, the water extracts with soil application increased the fresh weight of the shoots by 60–67% for the Hopyeong varieties and 60–65% for the Saenuri variety. Soil-applied ethanol extracts increased shoot fresh weight by 58–68% for Hopyeong and 55–63% for Saenuri. Foliar application of water extracts increased shoot fresh weight by 55–56% for Hopyeong and 55–60% for Saenuri. The ethanol extracts with foliar application increased shoot fresh weight by 55–65% in Hopyeong cultivars and almost 60% in Saenuri cultivars.

In both planting and extraction methods, the extracts of *P. guajava* and *A. sativum* showed the highest increase in shoot fresh weight in both cultivars compared with the control. The tested extracts had almost the same effect on the fresh weight of the shoots of both cultivars. The four extracts used in this study showed higher growth promotion rates than urea at 0.6% in transplanting tests; however, urea treatment had almost the same benefits as the tested plant extracts in direct seeding trials four weeks after treatment.

The results showed that the fresh weight of shoots increased by up to 48% with direct sowing and by up to 68% with transplanting. Therefore, the transplantation method was more suitable for determining the influence of plant extracts on rice growth. A total yield of 46–50% was obtained with the transplanting method compared with 13–38% with direct seeding [28]. Transplanted rice had a higher leaf chlorophyll content index, N concentration, total root length, and total root tip number than direct-seeded plants [29]. Planting methods can also influence early root development, allometric distribution of root and shoot biomass, fruit development, and marketable yields [30,31].

In addition, the highest shoot fresh weight of these rice varieties was obtained at 7 DAT in both water and ethanol treatments. Shoot fresh weight decreased slightly between 14 and 28 DAT. Similarly, [19] showed that extracts of Chinese chives, soybean leaves, and stems had a greater effect on the fresh weight of lettuce shoots when applied 7 days after sowing (DAS) than when applied at 14 and 21 DAS. Biostimulants can act directly on plant physiology and metabolism or by improving soil conditions [32].

### 2.4. Effect of Selected Water Extracts on Application Times in Various Growth Stages of Rice

**Figure 1:**
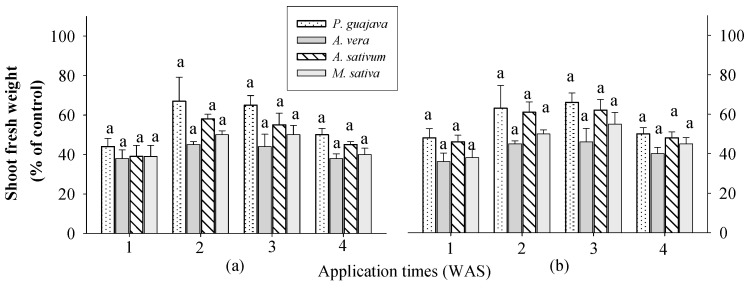
Effect of four water extracts on the shoot fresh weight of rice sprayed on different sowing times. (**a**) 0.1%; (**b**) 0.5% concentration. Means within bars followed by the same letters did not differ significantly in Tukey’s HSD at α = 0.05. WAS, week after sowing.

There were no significant differences between the crops and extracts at all application times (Figure 1). Shoot fresh weight increased by 28–48% when sprayed one week after sowing (WAS), 35–67% when sprayed 2 WAS, 36–66% when sprayed 3 WAS, and 26–50% when sprayed 4 WAS in rice. This means that all these extracts can increase the fresh weight of rice shoots at any growth stage and application time. The treated eggplants showed stimulatory responses in terms of growth and physiology depending on the repetition of the aqueous garlic bulb extract and the growth stage of the plants [20]. The highest shoot fresh weight values were observed in response to 0.1 and 0.5% *P. guajava* extract, followed by *A. sativum* extract in the rice plant. Garlic contains at least 33 sulfur compounds, enzymes, vitamins B and C, minerals (such as Na (sodium), K (potassium), Zn (Zinc), P (phosphorus), Mn (manganese), Mg (magnesium), Ca (calcium), and Fe (iron), carbohydrates, saponins, alkaloids, flavonoids, and free sugars (such as sucrose, fructose, and glucose) [33,34]. Guava leaves are rich in nitrogen, phosphorus, and potassium, depending on the month of growth [35], which supports the finding that guava leaf compost results in the best growth of the Temulawak plant (*Curcuma xanthorrhiza* Roxb). Therefore, it provides a balanced source of nutrients for plant growth.

### 2.5. Effect of Selected Water Extracts on Secondary Metabolites in Rice at Different Application Times

**Figure 2:**
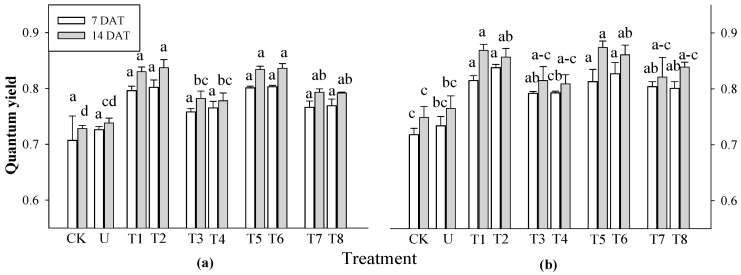
Effect of four water extracts on the quantum yield of rice sprayed on (**a**) 2 weeks after sowing (WAS); (**b**) 3 WAS. CK, control; T1 and T2, 0.1 and 0.5% of *P. guajava* extract; T3 and T4, 0.1 and 0.5% of *A. vera* extract; T5 and T6, 0.1 and 0.5% of *A. sativum* extract; T7 and T8, 0.1 and 0.5% of *M. sativa* extract. The parameter was recorded at 7 and 14 DAT. Means within bars followed by the same letters did not differ significantly in Tukey’s HSD at α = 0.05.

**Figure 3:**
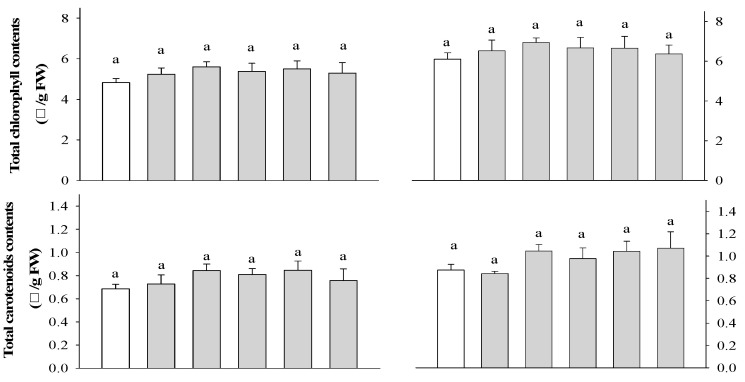

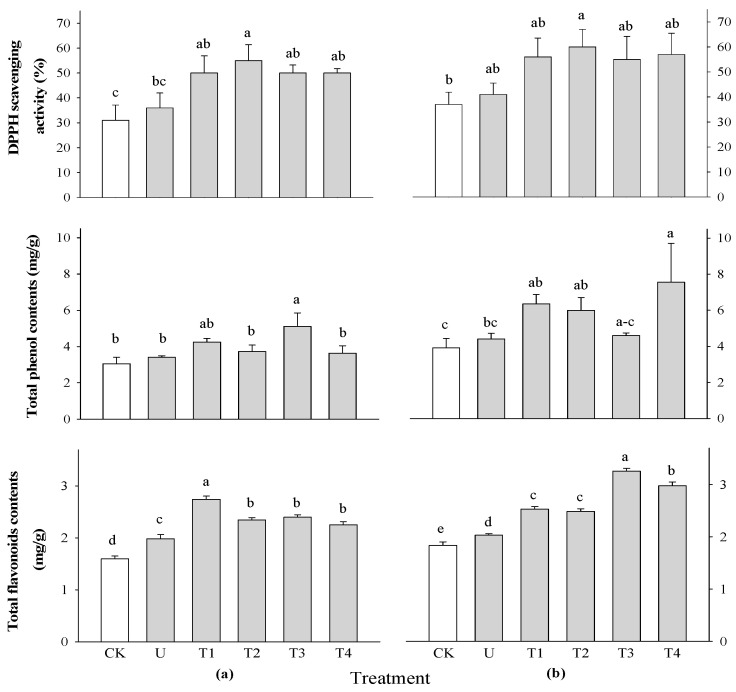
Effect of four water extracts on total chlorophyll and carotenoid contents, DPPH radical scavenging, total phenol, and flavonoid contents, in rice sprayed on (**a**) 2 weeks after sowing (WAS); (**b**) 3 weeks after sowing under greenhouse conditions. CK, control; U, urea; T1 and T2, 0.1 and 0.5% of *P. guajava*; T3 and T4, 0.1 and 0.5% of *A. sativum* extracts. Means within bars followed by the same letters did not differ significantly in the LSD test at α = 0.05.

Based on the results of the previous experiment, rice plants were selected to evaluate photosynthetic efficiency at 7 and 14 DAT. At 7 DAT, there was a significant difference in rice at 3 WAS for all treatments, but no significant difference was observed in rice at 2 WAS (Figure 2). At 14 DAT, there was a significant difference in the photosynthetic performance of the rice plants at 2 and 3 WAS. Photosynthetic performance increased in all extract treatments compared to that in the control and urea treatments. Among the extract treatments, the extracts of *P. guajava* and *A. sativum* resulted in a greater increase in photosynthesis in rice plants than the extracts of *A. vera* and *M. sativa*. These results strongly confirm that garlic root exudates increase chlorophyll content to enhance the absorption of light energy by tomatoes and peppers, resulting in improved photosynthetic rates [36]. None of the treatments did not differ significantly affected the total chlorophyll and carotenoid content of rice plants at any of the application times (Figure 3).

There was a significant difference in the DPPH radical scavenging activity of rice at 2 WAS; however, no significant difference was observed at 3 WAS (Figure 3). Moreover, the highest activity was found in rice treated with 0.5% *P. guajava* extract. The significant DPPH radical scavenging activity of *F. religiose* could be related to the presence of flavonoids and other polyphenols during extraction, as shown in the current study [37]. The antioxidant activity of the extracts evaluated by the DPPH assay was strongly correlated with the total phenolic and flavonoid content [38]. There was no significant difference in the total phenolic content of the rice plants (Figure 3), but there was a significant difference in the total flavonoid content of the rice plants at 2 and 3 WAS (Figure 3). The highest flavonoid content was found in the 0.1% *P. guajava* treatment at 2 WAS and the 0.1% *A. sativum* treatment at 3WAS in the rice plant. The phenolic and flavonoid groups may be largely responsible for the antioxidant activities of the selected plant extracts [39].

**Figure 4:**
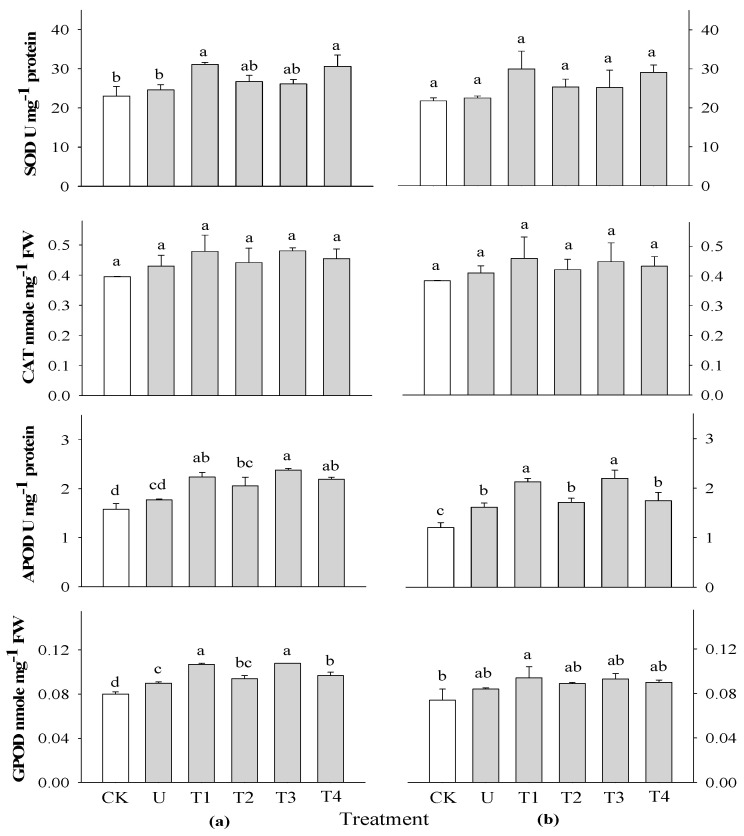
Effect of four water extracts on antioxidant activities in rice sprayed on (**a**) 2 weeks after sowing (WAS); (**b**) 3 weeks after sowing under greenhouse conditions. CK, control; U, urea; T1 and T2, 0.1 and 0.5% of *P. guajava*; T3 and T4, 0.1 and 0.5% of *A. sativum* extracts. Means within bars followed by the same letters did not differ significantly in the LSD test at α = 0.05.

The activities of SOD, CAT, GPOD, and APOD were investigated 2 and 3 WAS in rice plants using extracts of *P. guajava* and *A. sativum* at concentrations of 0.1 and 0.5% (Figure 4). There were no significant differences in SOD and CAT activities at all application times in rice plants compared with the control. This could not be related to SOD and CAT activities during the growth of rice. However, there were highly significant differences in APOD activity at different application times in rice plants compared with the control. The highest APOD activity was observed in rice treated with 0.1% *A. sativum* at both 2 and 3 WAS. There was no significant difference in the GPOD activity of rice at 3 WAS, but there was a significant difference in rice at 2 WAS when compared to the control. The highest GPOD was observed in the 0.1% *P. guajava* and *A. sativum* extract treatments at all application times. Spraying aqueous garlic extract stimulated the activity of antioxidant enzymes. Moderate application activated antioxidants and possibly reactive oxygen species (ROS), which led to enhanced plant growth; this was inhibited by a higher frequency of application [20]. An increase in SOD and POD activities during the early stages of plant growth suggests an increase in oxidative stress [40].

## 3. Materials and Methods

### 3.1. Preparation of Plant Materials and Extraction Methods

Rice seeds (cv. Hopyeong) were provided by Jeollanamdo Agricultural Research and Extension Service. For extraction, fifteen plant materials were collected from the fields and purchased through the Chonnam Hanyaknonghyup Cooperation (Table 4). The 17 plant extracts were prepared from 15 plant species, such as leaves of *Mentha arvense*, *Centella asiatica*, *Moringa oleifera*, *Vigna radiata*, *Vigna unguiculate*, *Psidium guajava*, *Aloe vera*, *Allium tuberosum*, aboveground plant parts of *Cyperus rotundus*, *Medicago sativa*, and *Perilla frutescens*, roots of *Rheum undulatum*, tubers of *Allium sativum*, leaves and stems of *Glycine max* (cv. Taegwang) as well as rice straw and hull (cv. Hopyeong).

Different plant species, including leaves, roots, tubers, and above-ground components, were dried, crushed, and extracted using ethanol, boiling water, and water [19]. For the water extract, 50 g of each agricultural material was homogenized in 1 L of distilled water for 24 h. For the ethanol extract, 1 L of ethanol was used for 24 h instead of distilled water. For the boiling water extract, 50 g of each agricultural material was dissolved in 1 L of distilled water, and the mixture was boiled at 100 °C for 30 min before being homogenized for 24 h. The extracts were filtered using a Whatman No. 1 polypropylene filter (45 × 50 cm) and then filtered through a double layer of Mira cloth (22–25 µm pore size). To ensure that the final concentration was 50%, each extract was evaporated using a rotary evaporator (N-1300, EYELA, Tokyo, Japan), and the concentrations were subsequently diluted with distilled water to obtain 0.05, 0.1, 0.5, and 1% concentrations. All extracts were stored in a refrigerator until further investigation.

### 3.2. Effect of Water Extracts from Various Agricultural Materials on Rice Growth

In the first phase of the experiment, rice (cv. Hopyeong) was used as a test crop, and different concentrations of 17 water extracts were evaluated (Table 2). To begin the experiment, rice seeds were soaked in distilled water for 24 h. Ten mL of 0.05, 0.1, 0.5, and 1% concentrations of the extracts were placed in a double layer of Whatman No.1 filter paper in 90 mm diameter Petri dishes. Distilled water was used as the control. Ten rice seeds were added to each petri dish after the extractions. The Petri dishes were maintained at room temperature for a week. After seven days of treatment, shoot and root lengths were measured. The seedling length was measured as the total length of the rice plant, including both the shoot and root portion

### 3.3. Effect of Selected Extracts on Rice Growth: Comparing Extraction, Spraying, and Planting Methods

Direct seeding and transplanting tests were performed under greenhouse conditions, and a Petri dish assay was also carried out in a growth chamber. The planting methods used included Petri dish bioassays, direct seedlings, and transplanting. *P. guajava*, *A. vera*, *A. sativum*, *M. sativum*, *A. tuberosum*, and *G. max* (stems and leaves) extracts were prepared using water, boiling water, and ethanol. The Petri dish assay was performed using the same procedure as the initial test.

The rice seeds were soaked in distilled water for 24 h. Commercial soil for rice nursery media (No. 1 Sunghwa, Boseong, South Korea) of approximately 130 g was filled into each pot (6 cm in height and 6 cm in diameter). For direct seeding, three rice seeds were placed in each pot after the soil had been filled. Three to five days later, selected 7 extracts were soil drenched with 10 mL per pot at concentrations of 0.05, 0.1, 0.5, and 1% into the soil. For transplanting, three rice seedlings were transplanted into each pot two weeks after sowing (three to four leaf stages). Seven selected extracts (10 mL per pot) with concentrations of 0.05, 0.1, 0.5, and 1% were applied either as a soil drench or via foliar application three to five days after transplanting. Distilled water was used as the control. Urea at 0.6% was used for comparison to evaluate the growth effectiveness of the extracts. Plants were watered as required. The pots were kept in a greenhouse for two weeks after the extract treatments. The greenhouse conditions included 14 h of light and 10 h of darkness, with a day/night temperature of 30 ± 2 °C/20 ± 3 °C, 70% relative humidity, and photosynthetically active radiation (PAR) of 500 µmol m^−2^ s^−1^ PAR. Plant height was measured at 7 and 14 day after transplantation, and shoot fresh weight was measured at 14 DAT.

### 3.4. Impact of Four Plant Extracts’ Persistence on Rice Growth

Rice seeds (cv. Hopyeong and Saenuri) were provided for research by the Jeollanamdo Agricultural Research and Extension Service. The two extraction methods used in these tests were water and ethanol, whereas the planting methods included direct seeding and transplanting. *P. guajava*, *A. vera*, *A. sativum*, and *M. sativum* were the four extracts chosen. Shoot fresh weight of rice was measured at weekly intervals on days 7, 14, 21, and 28 after treatment. All other procedures followed the same protocols described in Section 3.3.

### 3.5. Optimizing Application Timing of Selected Water Extracts in Rice Cultivation

The growth-promoting effects of four water extracts at concentrations of 0.1 and 0.5% were investigated when applied at different growth stages of rice. The application times were different for each growth stage (1, 2, 3, and 4 weeks after sowing). Shoot fresh weight was measured at 14 DAT. The other producers remained consistent with those described in Section 3.3.

### 3.6. Temporal Effects of Selected Water Extracts on Rice Secondary Metabolite Production

Three plants from each of the three replicates were sampled two weeks and three weeks after the application of the extracts. The leaf samples were immediately frozen in liquid nitrogen and stored at −80 °C for subsequent biochemical analysis.

#### 3.6.1. Determination of Quantum Yield, Total Chlorophyll and Carotenoid Contents

The chlorophyll α fluorescence of photosystem II (PSII), i.e., the quantum yield (Fv/Fm) of rice, was measured at 7 and 14 DAT. At 2 and 3 weeks after sowing (WAS), each plant received 10 mL of water extracts at concentrations of 0.1 and 0.5% administered via a soil drench. At 7 and 14 DAT, the second leaves of the rice plants were selected for determination using a portable pulse modulation fluorometer (Fluorpen FP10, Photon Systems Instruments, Drásov, Czech Republic). Before the measurements, the fronts were darkened for 15 min and adjusted to open all antenna pigments.

Chlorophyll and carotenoid analyses were performed according to a previously described method [19]. Seedling leaves (0.5 g) from each treatment group were ground in a 100% methanol solution. The extracts were centrifuged at 10,000× *g* for 3 min, and the absorbance of the supernatant was measured spectrophotometrically at 470, 652, and 665 nm. The chlorophyll and carotenoid contents were calculated using the following equation:

Chlorophyll a (C_a_) = 16.72 A_665.2_ − 9.16 A_65.4_

Chlorophyll b (C_b_) = 34.09 A_652.4_ − 15.28 A_665.2_

Total chlorophylls (C_a+b_) = 1.44 A_665.2_ + 24.93 A_652.4_

Total carotenoids (C_x+c_) = [1000 A_470_ − 1.63 C_a_ − 104.96 C_b_]/221

#### 3.6.2. Determination of DPPH Radical Scavenging Activity, Total Phenol, and Flavonoid Contents

DPPH radical scavenging activity and total phenol and flavonoid contents were determined according to a previously described method [41]. These activities were analyzed after 0.5 g of the dried plant samples were mixed with 10 mL of 99.9% ethanol, shaken at 120 rpm for 24 h at 27 °C in a shaking bath, and centrifuged at 13,000 rpm for 10 min (VS-24SMTI, high-speed refrigerated centrifuge, Vision Scientific Co., Ltd., Daejeon, Republic of Korea).

To measure DPPH radical scavenging activity, 0.1 mL of the extract, 0.5 mL of a 0.1 M acetate buffer solution (pH 5.5), 0.25 mL of 0.5 mM DPPH (2,2-diphenyl-1-picrylhydarzyl) and 0.4 mL of ethanol were mixed at room temperature and allowed to react for 30 min. The mixed solution was analyzed using a UV spectrometer at 517 nm (SPECTROstar Nana, BMG LABTECH, GmbH, Offenburg, Germany). Ascorbic acid was used as a positive control, and the DPPH radical scavenging activity of the extracts was calculated using the following formula:

DPPH radical scavenging activity (%) = [Ac − As/Ac] × 100

where Ac is the absorbance of the control, and As is the absorbance of the test sample [42].

To determine the total phenol content, 0.2 mL of the extract was mixed with 0.6 mL distilled water and 0.2 mL Folin-Denis reagent and then shaken for 5 min. After 5 min, 0.2 mL of Na_2_CO_3_ was added, and the mixed solution was allowed to stand for one h at room temperature and measured using a UV spectrophotometer at 640 nm (SPECTROstar Nana, BMG LABTECH, GmbH, Offenburg, Germany). The standard values were obtained with ferulic acid at 0–100 µg/mL, and the values of total phenol were calculated as a standard curve. All values are expressed as the mean (mg of ferulic acid equivalents per g of extracted sample).

To measure total flavonoid content, 0.2 mL of the extract was mixed with 0.45 mL of ethanol (95%), 10% AlCl_3_ 0.03 mL, 1 M potassium acetate 0.03 mL, and 0.79 mL distilled water. The mixed solution was then allowed to stand for 40 min at room temperature, and the absorbance was measured using a UV spectrophotometer at 640 nm (SPECTROstar Nana, BMG LABTECH, GmbH, Offenburg, Germany). The standard values were obtained with quercetic acid 0–5 µg/mL, and the values of total flavonoids were calculated as a standard curve. All values are expressed as the mean (mg of quercetinic acid equivalents per g of extracted sample).

#### 3.6.3. Determination of Antioxidant Activities

For enzyme extraction, frozen leaves (approximately 0.5 g) were homogenized with 3.75 mL of a 100 mM buffer solution (pH 7.5) containing 2 mM EDTA, 1% PVP, and 1 mM phenylmethylsulfonyl fluoride (PMSF, C_7_H_7_FO_2_S), crushed with a mortar and pestle and incubated at 14,000× *g* for 20 min in a refrigerated centrifuge (VS-24SMTI, high-speed refrigerated centrifuge, Vision Scientific Co., Ltd., Daejeon, South Korea).

Superoxide dismutase (SOD) activity was determined according to the method described in [43] with some modifications [44,45]. A 230 µL reaction mixture contained 1.26 mM NBT (Nitroblue tetrazolium), 260 mM riboflavin, 260 µM methionine, 2 mM EDTA, 200 mM phosphate buffer (pH 7.0), and 20 µL of enzyme extract in a total volume of 300 µL. The test tubes containing the mixture were placed under light at 78 mmol photons s^−1^ m^−2^ for 10 min, and the absorbance at 560 nm was recorded. A nonirradiated reaction mixture that did not develop color served as the control, and its absorbance was subtracted from A560 of the reaction solution. One unit of SOD activity was defined as the amount of enzyme required to cause 50% inhibition of the NBT reduction rate at 560 nm (SPECTROstar Nano, BMG LABTECH, GmbH, Offenburg, Germany).

The activities of catalase (CAT) and guaiacol peroxidase (GPOD) were measured using a previously described method [46,47]. For CAT activity, the decomposition of H_2_O_2_ was measured by the decrease in absorbance at 240 nm for 1 min (SPECTROstar Nano, BMG LABTECH, GmbH, Offenburg, Germany). The reaction mixture contained 150 µL of 200 mM phosphate buffer (pH 7.0), 15 µL of 15 mM H_2_O_2_, 135 µL of distilled water, and 5 µL of enzyme extract in a total volume of 300 µL, which initiated the reaction. For GPOD activity, the oxidation of guaiacol was measured by the increase in absorbance at 470 nm for 1 min (SPECTROstar Nano, BMG LABTECH, GmbH, Offenburg, Germany). The reaction mixture contained 5 µL of 200 mM guaiacol, 280 µL of 200 mM phosphate buffer (pH 7.0), and 15 µL of enzyme extract. The reaction was initiated using 5 µL of 40 mM H_2_O_2_.

Ascorbate peroxidase (APOD) activity was assayed according to the method of [48], using the initial rate of decrease in ascorbate concentration, as measured by its absorbance at 290 nm. The reaction mixture contained 150 µL of 200 mM phosphate buffer (pH 7.0), 30 µL of 5 mM ascorbate, 12 µL of 20 mM H_2_O_2_, 30 µL distilled water, and 15 µL enzyme extract in a total volume of 300 µL. The reaction was initiated by adding H_2_O_2,_ and the change in absorbance was measured using a UV spectrophotometer (SPECTROstar Nano, BMG LABTECH, GmbH, Offenburg, Germany).

### 3.7. Experimental Design and Statistical Analysis

The experiment was designed with three replicates in a completely randomized design with a factorial layout. Multiple comparisons were conducted to evaluate the differences in the variables between the factors. Significant differences were evaluated using an analysis of variance (ANOVA) with a statistical computer program (Statistix version 8.0 software). When there was a significant difference, the means were separated using Tukey’s honestly significant difference (HSD) and Least significant difference (LSD) tests at α = 0.05.

## 4. Conclusions

This study found that plant extracts, especially water extracts, were more effective in promoting rice growth when applied as soil-applied than foliar-application. Transplanting methods were better suited for determining the influence of plant extracts on rice growth. All extracts increased the fresh weight of shoots in rice plants, with concentrations of 0.1 and 0.5% achieving the best growth promotion rates. The effectiveness of the extracts was maintained even after 14 days of treatment. Therefore, all extracts can be sprayed on rice plants at two- or three-week intervals. This study showed that photosynthesis and antioxidant activity may play crucial roles in plant growth. It means that all metabolites contribute to the process of photosynthesis and provide a source of nutrients, and all of this contributes to the main compounds in the plant and improves its physiological characteristics. This study concluded that *Psidium guajava*, *Aloe vera*, *Allium sativum*, *and Medicago sativa* extracts can be used to promote plant growth in organic farming.

## Figures and Tables

**Table 1:** Growth promotion rate (% of control) of water extracts of various plant materials on rice seedlings. The parameter was recorded at 7 days after treatment in a Petri dish bioassay.

Growth Promotion Rates (% of Control)	Shoot Length	Root Length	Seedling Length (Shoot + Root)
10–20	*Mentha arvensis* *Rheum undulatum* *Cyperus rountus* *Perilla frutescens* *Oryza sativa* (hull) *Vigna unguiculata*		*Vigna unguiculata*
21–30	*Centella asiatica* *Allium sativum* *Medicago sativa* *Moringa oleifera* *Vigna radiata*	*Mentha arvensis* *Cyperus rountus* *Perilla frutescens* *Oryza sativa* (straw) *Vigna radiata* *Vigna unguiculata*	*Mentha arvensis* *Cyperus rountus* *Centella asiatica* *Perilla frutescens* *Oryza sativa* (straw) *Oryza sativa* (hull) *Vigna radiata*
31–40	*Allium tuberosum* *Oryza sativa* (straw) *Glycine max* (leaves)	*Rheum undulatum* *Centella asiatica* *Aloe vera* *Oryza sativa* (hull) *Moringa oleifera*	*Rheum undulatum* *Aloe vera* *Medicago sativa* *Moringa oleifera* *Allium tuberosum*
41–50	*Psidium guajava* *Aloe vera* *Glycine max* (stem)	*Medicago sativa* *Allium tuberosum* *Glycine max* (leaves) *Glycine max* (stem)	*Psidium guajava* *Allium sativum* *Glycine max* (stems) *Glycine max* (leaves)
51–60		*Psidium guajava*	
61–70		*Allium sativum*	

**Table 2:** Mean values of shoot, root, and seedling length of rice were applied by selected plant extracts using different extraction methods in a Petri dish bioassay.

	Shoot Length (cm)	Root Length (cm)	Seedling Length (cm)
Extract			
Water	3.60 ^b^	5.70 ^a^	9.29 ^a^
Boiling water	3.13 ^c^	5.07 ^c^	8.19 ^c^
Ethanol	3.79 ^a^	5.26 ^b^	9.05 ^b^
HSD_0.05_	0.10	0.10	0.15
Concentration (%)			
0 (control)	3.05 ^b^	4.14 ^d^	7.46 ^d^
0.05	3.80 ^a^	5.96 ^a^	9.76 ^a^
0.1	3.85 ^a^	5.89 ^ab^	9.74 ^a^
0.5	3.70 ^a^	5.76 ^b^	9.46 ^b^
1	3.15 ^b^	4.66 ^c^	7.81 ^c^
HSD_0.05_	0.16	0.15	0.23
Treatment			
*P. guajava*	3.53 ^a^	5.54 ^a^	9.07 ^a^
*A. vera*	3.50 ^a^	5.14 ^cd^	8.64 ^b^
*A. sativum*	3.55 ^a^	5.05 ^d^	8.59 ^b^
*M. sativa*	3.53 ^a^	5.48 ^a^	9.02 ^a^
*A. tuberosum*	3.51 ^a^	5.26 ^bc^	8.83 ^ab^
*G. max* (leaves)	3.39 ^a^	5.45 ^ab^	8.84 ^ab^
*G. max* (stem)	3.50 ^a^	5.45 ^ab^	8.94 ^a^
HSD_0.05_	0.20	0.20	0.29
Pr ≥ F			
E	***	***	***
C	***	***	***
T	ns	***	***
E × T × C.	***	***	***
CV%	10.56	6.87	6.19

Means in the same column followed by the same letter are not significantly different from each other at the 5% level of Tukey’s HSD test. Seedling length is a combination of shoot and root length. E: extracts. T: treatments. C: concentrations. ***: significant at 0.001 level. ns: non-significant.

**Table 3:** Mean values of plant height and shoot fresh weight of rice were applied by selected plant extracts using different extraction methods compared with direct seeding and transplanting tests.

	PH at 7 DAT (cm)	PH at 14 DAT (cm)	SFW at 14 DAT (g/3 Plants)
Planting method			
Direct seeding	4.34 ^b^	14.59 ^b^	0.181 ^b^
Transplanting	26.68 ^a^	35.99 ^a^	0.974 ^a^
HSD_0.05_	0.142	0.153	0.018
Extract			
Water	15.78 ^a^	26.88 ^a^	0.682 ^a^
Boiling water	15.76 ^a^	23.64 ^c^	0.607 ^b^
Ethanol	14.98 ^b^	25.36 ^b^	0.442 ^c^
HSD_0.05_	0.208	0.224	0.026
Concentration (%)			
0 (control)	12.945 ^d^	21.15 ^d^	0.456 ^d^
0.6 (Urea)	13.73 ^c^	23.18 ^c^	0.501 ^c^
0.05	16.63 ^ab^	26.99 ^a^	0.653 ^a^
0.1	16.74 ^a^	27.27 ^a^	0.637 ^ab^
0.5	16.68 ^ab^	25.46 ^a^	0.622 ^ab^
1	16.35 ^b^	26.21 ^b^	0.596 ^b^
HSD_0.05_	0.358	0.385	0.044
Treatment			
*P. guajava*	15.71 ^a^	25.49 ^a^	0.585 ^a^
*A. vera*	15.32 ^ab^	25.15 ^ab^	0.594 ^a^
*A. sativum*	15.58 ^ab^	25.46 ^a^	0.582 ^a^
*M. sativa*	15.65 ^ab^	25.46 ^a^	0.589 ^a^
*A. tuberosum*	15.45 ^ab^	25.21 ^ab^	0.566 ^a^
*G. max* (leaves)	15.56 ^ab^	25.31 ^ab^	0.556 ^a^
*G. max* (stem)	15.29 ^b^	24.96 ^b^	0.569 ^a^
HSD_0.05_	0.400	0.430	0.049
Pr ≥ F			
P	***	***	***
E	***	***	***
T	**	***	ns
C	***	***	***
P × A × E × T × C	ns	***	ns
CV%	7.43	4.89	24.48

Means in the same column followed by the same letter are not significantly different from each other at the 5% level of Tukey’s HSD test. A: application methods. E: extracts. T: treatments. C: concentrations. PH: plant height. SFW: shoot fresh weight. DAT: days after treatment with extracts. ***: significant at 0.001 level. **: significant at 0.01 level. ns: non-significant.

**Table 4:** Plant materials used in this study.

Scientific Name	Common Name	Family Name	Parts Used
*Allium sativum*	Garlic	Amaryllidaceae	Tubers
*Allium tuberosum*	Chinese (garlic) chives or Chinese leeks	Amaryllidaceae	Leaves
*Centella asiatica*	Pennywort (Gotu Kola)	Apiaceae	Leaves
*Aloe vera*	Aloe	Asphodelaceae	Leaves
*Cyperus rountus*	Nutgrass	Cyperaceae	Aboveground plant parts
*Mentha arvensis*	Wild Mint	Lamiaceae	Leaves
*Perilla frutescens*	Chinese basil, wild basil, perilla mint	Lamiaceae	Aboveground plant parts
*Medicago sativa*	Alfalfa	Leguminosae	Aboveground plant parts
*Vigna radiata*	Mung bean	Leguminosae	Leaves
*Vigna unguiculata*	Cowpea	Leguminosae	Leaves
*Glycine max*	Soybean	Leguminosae	Leaves
*Glycine max*	Soybean	Leguminosae	Stems
*Oryza sativa*	Rice straw	Gramineae	Straws
*Oryza sativa*	Rice hull	Gramineae	Hulls
*Moringa oleifera*	Drumstick	Moringaceae	Leaves
*Psidium guajava*	Guava	Myrtaceae	Leaves
*Rheum undulatum*	-	Polygonaceae	Roots

## Data Availability

Data are contained within the article.
